# The Prognostic Value of the Circulating Tumor Cell-Based Four mRNA Scoring System: A New Non-Invasive Setting for the Management of Bladder Cancer

**DOI:** 10.3390/cancers14133118

**Published:** 2022-06-25

**Authors:** Consuelo Amantini, Federica Maggi, Jacopo Adolfo Rossi de Vermandois, Marilena Gubbiotti, Antonella Giannantoni, Ettore Mearini, Massimo Nabissi, Daniele Tomassoni, Giorgio Santoni, Maria Beatrice Morelli

**Affiliations:** 1 School of Biosciences and Veterinary Medicine, University of Camerino, 62032 Camerino, Italy; federica.maggi@unicam.it (F.M.); daniele.tomassoni@unicam.it (D.T.); 2 Urologic and Andrologic Clinics, University of Perugia, 05100 Perugia, Italy; ja.rossidevermandois@aospterni.it (J.A.R.d.V.); ettore.mearini@unipg.it (E.M.); 3 Department of Urology, San Donato Hospital, 52100 Arezzo, Italy; marilena.gubbiotti@uslsudest.toscana.it; 4 Department of Medical and Surgical Sciences, University of Siena, 53100 Siena, Italy; antonella.giannantoni@unisi.it; 5 Neurosciences, Functional and Surgical Urology Unit, Santa Maria alle Scotte Hospital, 53100 Siena, Italy; 6 School of Pharmacy, University of Camerino, 62032 Camerino, Italy; massimo.nabissi@unicam.it (M.N.); giorgio.santoni@unicam.it (G.S.)

**Keywords:** circulating tumor cells, bladder cancer, recurrence, mRNA scoring system, TRPM4

## Abstract

**Simple Summary:**

Bladder cancer with similar diagnosis based on traditional classification exhibits different behaviors and therapeutic outcomes. Thus, circulating tumor cells (CTCs) represent a more accurate approach to investigate bladder cancer features. Our results demonstrate that risk score based on EGFR, TRPM4, TWIST1, and ZEB1 four-gene signature in CTCs is markedly and undoubtedly associated with recurrence, suggesting an innovative and non-invasive strategy to manage both non muscle invasive and muscle invasive bladder cancer progression without the necessity of repetitive and onerous cystoscopies.

**Abstract:**

Bladder cancer (BC) is one of the most expensive lifetime cancers to treat because of the high recurrence rate, repeated surgeries, and long-term cystoscopy monitoring and treatment. The lack of an accurate classification system predicting the risk of recurrence or progression leads to the search for new biomarkers and strategies. Our pilot study aimed to identify a prognostic gene signature in circulating tumor cells (CTCs) isolated by ScreenCell devices from muscle invasive and non-muscle invasive BC patients. Through the PubMed database and Cancer Genome Atlas dataset, a panel of 15 genes modulated in BC with respect to normal tissues was selected. Their expression was evaluated in CTCs and thanks to the univariate and multivariate Cox regression analysis, EGFR, TRPM4, TWIST1, and ZEB1 were recognized as prognostic biomarkers. Thereafter, by using the risk score model, we demonstrated that this 4-gene signature significantly grouped patients into high- and low-risk in terms of recurrence free survival (HR = 2.704, 95% CI = 1.010–7.313, Log-rank *p* < 0.050). Overall, we identified a new prognostic signature that directly impacted the prediction of recurrence, improving the choice of the best treatment for BC patients.

## 1. Introduction

Bladder cancer (BC) is the tenth most common cancer worldwide and its incidence is gradually increasing, particularly in developed countries [1]. The American Cancer Society estimates that for 2022, there will be about 81,180 new cases and 17,100 deaths for BC in the United States. BC is divided into non-muscle-invasive bladder cancer (NMIBC) and muscle-invasive bladder cancer (MIBC). It is one of the most expensive lifetime cancers to treat because of the high recurrence rate, repeated surgeries, and long-term cystoscopic monitoring and treatment [2]. The highest cost is related to the management of NMIBC patients, which is around 70% of BC [3]. At the time of presentation, most are stage Ta tumors that do not penetrate the epithelial basement membrane and about 20% are stage T1, invading the submucosa and with high risk of progression to muscle invasion [4]. T1 has an almost 40% rate of recurrence and 20% of progression at 5 years, despite primary intravesical therapy with Bacillus Calmette–Guérin [4]. As has been widely demonstrated, traditional classification systems based on pathological parameters cannot fully reflect the “intrinsic characteristics” of BC [5]. In fact, the progression of BC differs seriously among individuals with similar pathological staging and grading and, consequently, the selection of the optimal monitoring and treatments based on traditional classification is not always in line with the patient’s actual clinical situation. 

Several efforts are being made to improve the understanding of these types of tumors with the aim to better stratifying patients to reduce or eliminate the risk of recurrence and progression. Primary BCs are molecularly and clinically heterogeneous [6,7,8,9,10,11]. Therefore, the characterization of primary tumors might not be enough and the establishment of a more reasonable specimen collection and management procedure should be the first step to ensure the accuracy of molecular typing results [5]. Thus, by using urine and blood liquid biopsy platforms, a new direction in the decision-making process for targeted BC therapies represents a fundamental goal [12].

Scientific discoveries have demonstrated the relevance of circulating tumor cells (CTCs). CTCs are cancer cells that have been detached from the primary tumor mass and are wiped out by the circulatory or lymphatic system [13]. In several cancer types, the enumeration of CTCs from the peripheral blood is recognized as a predictive indicator of cancer prognosis [14,15,16,17,18,19,20,21,22,23]. Regarding BC, in a cohort of T1 BC patients, the presence of CTCs correlated with a shorter time to first recurrence and time to progression [24,25]. Nevertheless, there was a non-statistical difference in the number of preoperative CTCs between the NMIBC and MIBC patients, suggesting that further analysis is needed [26]. Since the isolation of CTCs suggests that invasive treatment gives rise to the possibility of spreading into the systemic circulation [27,28,29], the discovery of diagnostic and prognostic molecular information from CTCs is critical [30]. 

Detaching from the primary tumors, CTCs lose the biochemical and biophysical signaling from the extracellular matrix and, consequently, start to be subjected to several changes. The shape changes that occur in CTCs are driven by cytoskeletal remodeling and the resulting stretching of chromatin may influence gene expression [31,32]. In addition, tension in the cytoskeletal system is likely to affect mechanosensitive components such as the nuclear lamina and ion channels on the cell membrane [33,34,35]. Thus, the transition from primary tumor cells to CTCs affects the signaling pathway and gene expression in these cells [36]. 

Therefore, the aim of this paper was to identify a specific gene signature based on CTC analysis that is able to molecular subtype patients to better guide therapeutic decision by using a non-invasive sampling. Starting from the knowledge of the literature, we focused the analysis on a selected panel of genes involved in BC growth and progression that also included members of the transient receptor potential channels (TRPs). These are calcium permeable channels, localized both in the plasma membrane and in the intracellular compartment known to be responsible for tumorigenesis and progression through the regulation of tumor invasion and metastasis in several cancers [37]. 

## 2. Materials and Methods

### 2.1. Identification of Previously Published Biomarker Candidates

A NCBI PubMed (http://www.pubmed.com/, accessed on 10 September 2018) search conducted in 2018 using the keywords ‘human bladder cancer’ combined with ‘mRNA biomarkers’ and ‘overall survival’ resulted in 141 hits and in 86 hits for ‘genitourinary cancer’ with ‘transient receptor potential channel’. All research articles were written in English, dating back to the maximum year of publication of 2010, and the evaluated biomarkers involved in bladder cancer growth and progression were included. In vitro and in vivo studies concerning human samples were also included. Studies with limited information or with an inaccurate number of samples were excluded. The validity of the articles, after applying the inclusion and exclusion criteria, were assessed by three researchers (C.A., M.B.M., and F.M.).

The Cancer Genome Atlas (TCGA) and Genotype-Tissue Expression (GTEx) datasets were screened and the accessible transcriptomic data of patients diagnosed with BC were obtained (http://www.gepia.2cancer-pku.cn, accessed on 22 October 2018) with OS available for 404 patients. 

### 2.2. Study Patients

This translational study included 60 patients with NMIBC or MIBC, hospitalized before transurethral resection of bladder (TURB), at the Urologic and Andrologic Clinics, University of Perugia, Perugia, Italy. Moreover, to improve the clarity/soundness of the results, the exclusion criteria were: another histological type of cancer, had received neoadjuvant or adjuvant chemotherapy, had a medical history of the same or another type of cancer, and had received a T0 stage classification. Enrolled patients had a clinical and radiological evidence of BC and did not undergo systemic/locally therapy before and after the TURB and the blood sample collection. The diagnosis was confirmed by histological analysis. The patient data regarding the age, tumor stage, histological type, regional lymph node involvement, and number of metastatic sites were also tabulated and statistically analyzed (Table 1). Local was used to define the locoregional recurrence at the surgical field, and distant to describe the presence of metastatic disease outside the urinary tract and out of the locoregional surgical field. Imaging techniques such as computed tomography were applied to assess the presence of metastasis. No patient died during the investigation period nor underwent a cystectomy.

Blood samples from 53 BC patients were collected and analyzed between March 2018 and March 2020. Healthy donors (*n* = 5) were age-matched men without BC who were recruited for normal tissue specimens according to the Ethics Committee of the University of Perugia-approved protocol. Moreover, fresh samples (*n* = 8) of primary tumors were recovered for comparative analyses.

The present study was approved by the Ethical Committee CEAS UMBRIA (Ethic Committee approval code: URO009-3171/18). The use of patient data and CTCs for research purposes at the University of Perugia were executed pursuant to the Italian legislation and international standards. Anonymous written informed consent was obtained from each participant or their guardians prior to the study enrolment.

### 2.3. Patient Samples

For CTC detection, 7 mL of peripheral blood from each patient was collected into Vacutainer^®^ Blood Collection Tubes EDTA (BD Biosciences, Franklin Lake, NJ, USA) on the day of surgery. Blood was obtained at the middle of vein puncture after the first 2 mL of blood was discarded to avoid contamination by normal epithelial cells. Peripheral blood was immediately processed within 3 h by means of ScreenCell devices (Sarcelles, France), as previously described [38], to isolate the CTCs using a size-based method already used in rare tumors [39] as well as in more common tumors including BC [40]. 

Seven blood samples from healthy donors were obtained from the blood bank to establish the normal expression of each marker in the white blood cells as previously described [38]. 

In addition, fresh BC tissue samples (biopsies), obtained during TURB from eight patients, were used in the study.

### 2.4. RNA Extraction

A Single Shot Cell Lysis Kit (BioRad, Hercules, CA, USA) was employed to extract the total RNA according to the protocol. Messenger RNA from biopsies was extracted by the RNeasy Mini Kit (Qiagen, Milan, Italy). Messenger RNAs from five human normal bladder samples (NHB) were purchased from AMS Biotechnology (Abigdon, UK).

### 2.5. Reverse Transcription

The iScript Advanced cDNA Synthesis Kit (BioRad) was employed to retrotranscribe RNA and the SSOADvanced PreAmp Kit and PrimePCR PreAMP Assays (BioRad) were used to preamplify the cDNA including all of the primers used in the gene expression analysis.

### 2.6. Digital Droplet PCR (ddPCR)

The ddPCR was performed by using the ddPCRSupermix for Probes (No dUTP) (BioRad) and the specific PrimePCR™ ddPCR™ Expression Probe Assays conjugated with the FAM or HEX fluorescent dyes (the same pool used in the preamplification step) (BioRad). The following target genes were analyzed: Baculoviral IAP Repeat Containing 5 (BIRC5), cadherin 11 (CDH11), epidermal growth factor receptor (EGFR), epithelial cell adhesion molecule (EPCAM), keratin 18 (KRT18), secreted phosphoprotein 1 (SPP1), transient receptor potential cation channel subfamily A member 1 (TRPA1), subfamily C Member 1 (TRPC1), member 3 (TRPC3), member 6 (TRPC6), subfamily V member 6 (TRPV6), subfamily M member 4 (TRPM4), tenascin (TNC), Twist family BHLH transcription factor 1 (TWIST1), vascular endothelial growth factor A (VEGFA), vimentin (VIM), and zinc finger E-box binding homebox 1 (ZEB1). QuantaSoft Software (BioRad) was used to analyze the data, expressed as copies/µL and normalized to a ß-actin concentration. ddPCR analysis was also performed to analyze the CD3D, CD19, CD45, ICAM1, CD41, CD235a, and Beta2-microglobulin gene expression in BC samples, given that some of the aforementioned transcripts could also be expressed, although at low levels, in normal blood cells. The results obtained from BC patients were compared with those obtained from the peripheral blood mononuclear cells from healthy donors and used them as the negative threshold. Gene expression was shown as fold respect to a calibrator (=1). 

### 2.7. Immunofluorescence and Confocal Microscopy

CTCs were fixed in 4% paraformaldehyde, permeabilized with phosphate buffered saline (PBS) plus 0.2% Triton X-100 and blocked for 60 min with 1% bovine serum albumin in PBS. Mouse anti-human pan-cytokeratin (C11) Ab (1:50, sc-8018, Santa Cruz Biotechnology, Heidelberg, Germany), anti-human CD45 Ab (1:50, #13917, Cell Signaling Technology, Danvers, MA, USA), anti-human TWIST1 Ab (1:50, sc-81417, Santa Cruz Biotechnology), anti-human EGFR (1:50, sc-373746, Santa Cruz Biotechnology), anti-human TRPM4 (1:50, HPA041169, Sigma Aldrich, St. Louis, MO, USA), and anti-human ZEB1 (1:50, sc-515797, Santa Cruz Biotechnology), followed by corresponding IgG Abs (Alexa Fluor^®^ 594, 1:100, Abcam, Cambridge, UK) were used to stain the CTCs. PureBluTM DAPI (#1351303, BioRad) labeled the nuclei. C2 Plus confocal laser scanning microscope (Nikon Instruments, Firenze, Italy) and NIS Element Imaging Software (Nikon Instruments) were used for the acquisition and processing of data. Epithelial CTCs required having a DAPI-positive nucleus with a diameter of 4 μm, cytokeratin staining surrounding 50% of the nucleus, and no CD45 expression.

### 2.8. InCTC Assay

The InCTC assay was performed starting from blood diluted with PBS plus 2% fetal bovine serum (FBS), processed with Ficoll-Paque premium gradient medium (GE Healthcare, Milan, Italy). The monolayer containing the CTCs and leukocytes was then collected and washed with RPMI medium without serum. A micro-Boyden transwell chamber (Greiner Bio One, Cassina de Pecchi, Italy) with 8-μm-diameter pore inserts coated with Matrigel was exploited to assess the migration ability of the CTCs. The CTCs were seeded onto the upper chamber in the medium without serum, while 10% FBS was added to the lower chamber. The chambers were then incubated in a humidified incubator with 5% CO_2_. After 24 h, the inserts were washed with PBS, fixed with ice-cold methanol, rinsed with PBS, and swabbed with a cotton swab to remove the non-migrated cells. Toluidine blue staining was performed to visualize the CTCs with light microscopy analysis, with DAPI and anti-pan CK for confocal analysis. The T24 cell line was used as a positive control. The T24 cell line, cultured in complete RPMI 1640 medium (10% FBS plus 1% penicillin-streptomycin) and grown at 37 °C in a 5% CO_2_ incubator, was used as the positive control.

### 2.9. Protein–Protein Interaction (PPI) Network Analysis

The search tool for retrieval of interacting genes (STRING) (https://string-db.org, accessed on 22 October 2018) database, based on known and predicted PPIs, was employed to seek potential interactions between the markers [41]. Text mining, experiments, databases, co-expression, species limited to “*Homo sapiens*”, and an interaction score >0.7 were considered as active interaction sources and applied to construct the PPI networks. The PPI network was visualized by Cytoscape software version 3.6.1. (https://cytoscape.org/, accessed on 22 October 2018) In the networks, proteins are schematized as nodes and interactions as edges. 

### 2.10. Prognostic Signature

The univariate Cox proportional hazard regression model was used to assess the association of gene expression in CTC with the recurrence free survival (RFS) of patients included in the cohort. Genes with *p* ≤ 0.05 were considered statistically significant. Using the regression coefficients from the Cox regression analysis of significant genes, we calculated a risk score for each patient based on their individual expression levels. In order to improve reliability, patients were stratified using the median risk score as a cutoff value, and the BC patients were classified into high- (1) and low-risk (0) groups, and Kaplan–Meier and receiver operating characteristic (ROC) analyses were performed.

### 2.11. Statistical Analysis

The quantitative variables are expressed as the mean values ± standard deviation (SD) or as the median values (interquartile range). Qualitative variables are expressed as absolute and relative frequencies. For the comparisons of the proportions, categorical data were tested by the Fisher’s exact test. 

The differential expression gene (DEG) analysis was performed by applying the Mann–Whitney U *t*-test. The correlation matrix was analyzed by the Spearman correlation test.

The Cox proportional hazard regression model was used to perform the univariate and multivariate survival analyses. Only statistically significant variables in the univariate analysis were incorporated into the risk score equation. To assess whether the risk score was independent of other clinical characteristics, multivariable Cox regression analysis and stratification analysis were used.

The differences in the recurrence time of BC patients stratified by low- and high-risk according to gene expression in CTCs was assessed by the Kaplan–Meier method. The RFS was considered as the time from the TURB surgery to the date of the first recurrence or, if the recurrence of tumor was not present, the date of the last follow-up. The statistical significance of the observed differences between groups was determined by the log-rank test. 

The grading variable was dichotomized by the time-dependent ROC curve analysis. The cutoff point estimated by the ROC analysis indicates the distinguishing characteristic of the grading variable used to classify participants into the recurrence group. The accuracy of the dichotomous results was calculated through the area under the ROC curve (AUC). 

All testing was carried out using GraphPad Prism 9.1 software (GraphPad Software, La Jolla, CA, USA) or SPSS (version 26.0; Chicago, IL, USA) or R (version 4.1.2, Auckland, NZ, USA). All *p*-values were two-sided, and *p* ≤ 0.05 was considered to indicate statistical significant.

## 3. Results

### 3.1. Selection of Previously Described BC Biomarker Candidates

**Figure 1:**
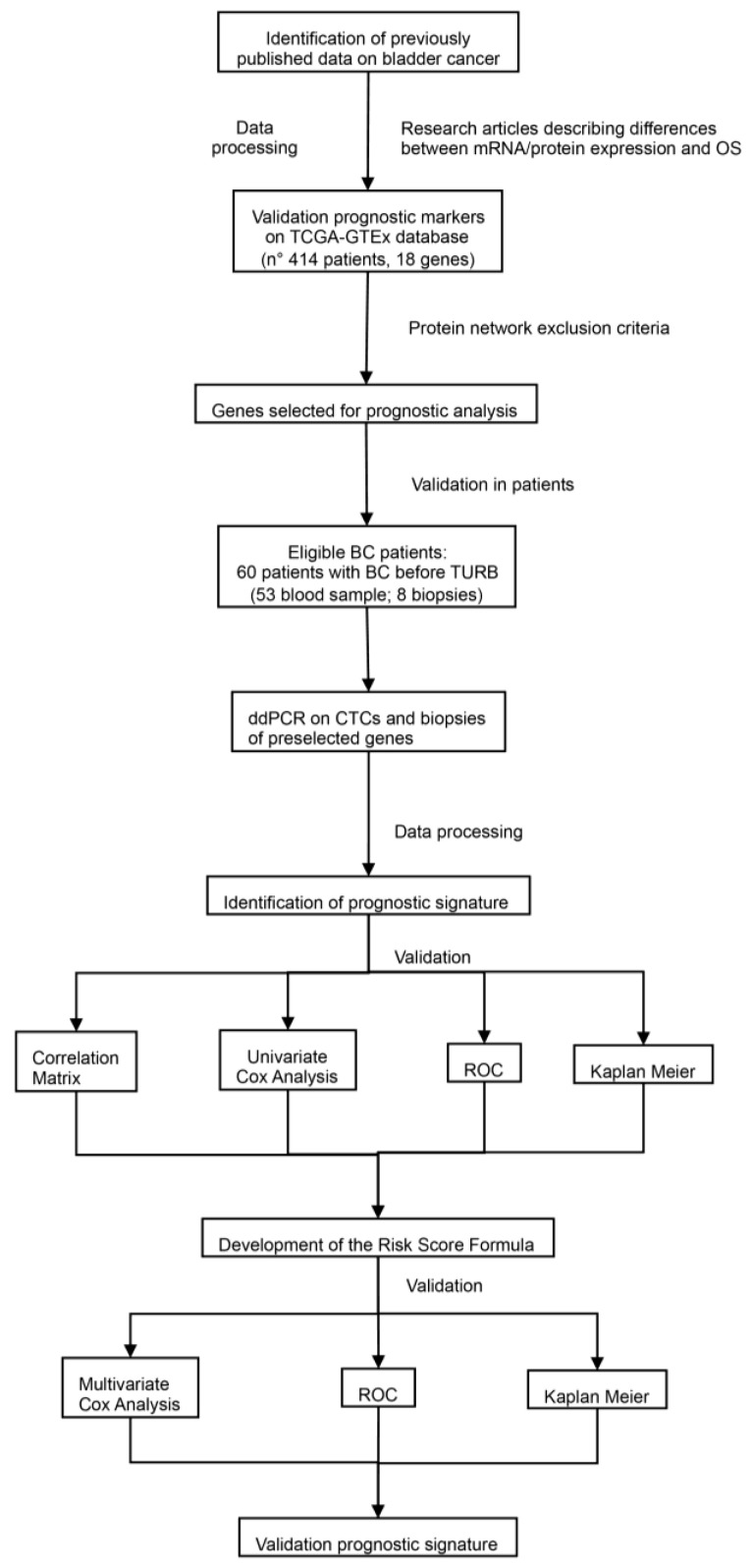
The flowchart of the data processing, analysis, and validation in the current study.

Analysis of the PubMed search highlighted several bladder-specific transcripts implicated in BC growth and metastasis. Among them, we selected BIRC5, CDH11, EGFR, EPCAM, KRT18, SPP1, TRPA1, TRPC1, TRPC3, TRPC6, TRPV6, TRPM4, TWIST1, TNC, UPK2, VEGFA, VIM, and ZEB1 as potential biomarker candidates in our investigation to develop a prognostic signature model. Figure 1 illustrates the work flow diagram of this study.

**Figure 2:**
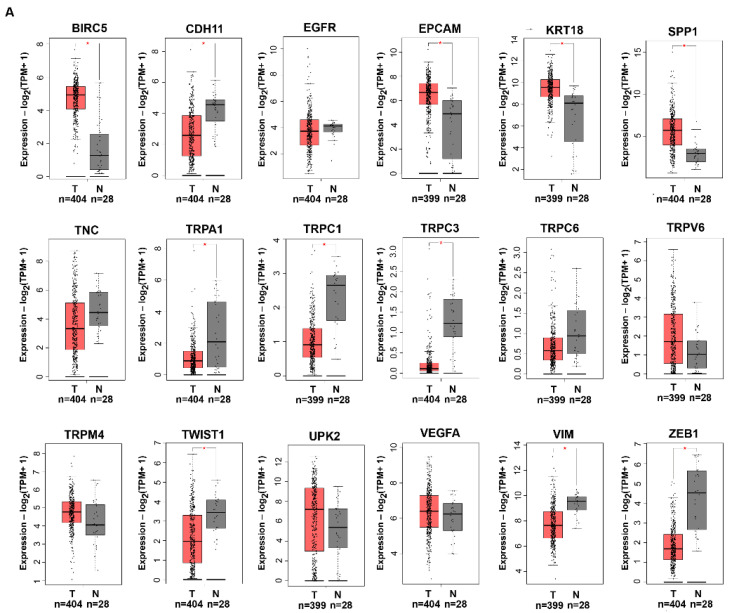

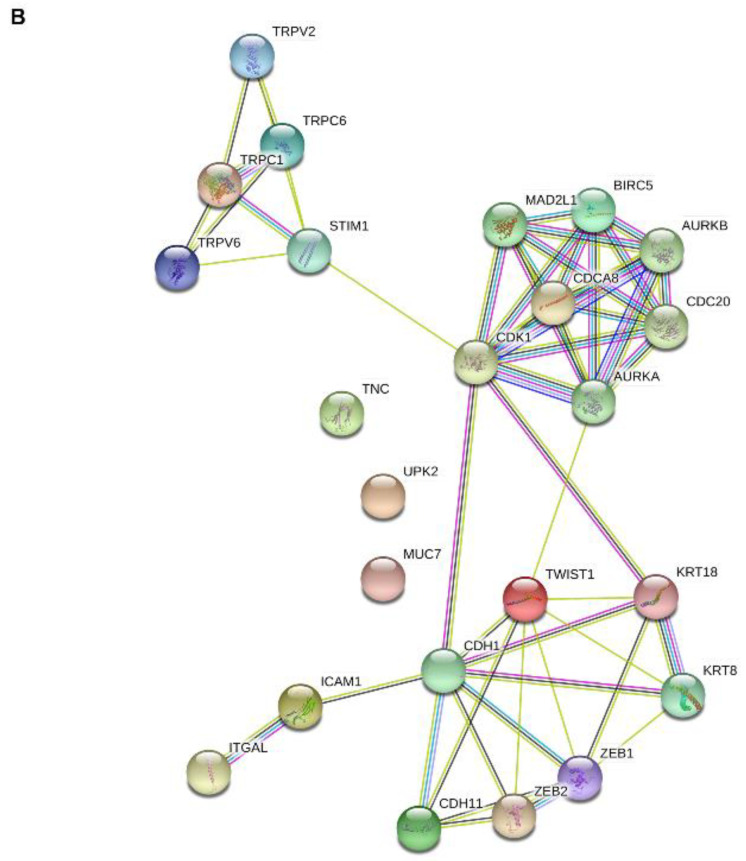
The selection of potential biomarkers. (**A**) The comparison of the mRNA expression level of the selected potential biomarker candidates between the normal and tumor tissues from thee TCGA-GTEx database using GEPIA. T = Tumor, N = normal bladder. (**B**) The protein–protein interaction network derived from STRING connects the selected markers (PPI enrichment, *p* value < 1.0 × 10^−16^).

First, through the gene expression profiling interactive analysis (GEPIA2), we analyzed the different expression of these candidates between the normal and tumor bladder tissue (Figure 2A). Results showed that the BIRC5, EPCAM, KRT18 and SPP1 genes were upregulated, and CDH11, TRPA1, TRPC1, TRPC3, TWIST1, VIM, and ZEB were downregulated whereas EGFR, TNC, TRPC6, TRPV6, TRPM4, UPK2, and VEGFA were not differently expressed in BC with respect to the normal tissue. Then, we constructed the network connecting the selected key genes to the predicted functional associations (Figure 2B). The PPI network was visualized in the form of a graph network. Most of the proteins were functionally associated, while UPK2 was completely not connected to the network and so it was not included in the successive analyses. Interestingly, the cluster of TRP cation channels was associated with the rest of the group by the gene PLCG1, which codifies for a calcium-dependent enzyme involved in signal transduction and in the regulation of TRP activity [42,43,44].

### 3.2. Isolation and Gene Expression Profiling of CTCs from BC Patients

It is well-known that CTCs acquire different features with respect to the primary tumor cells by changing gene expression [36]. Thus, we evaluated the gene expression levels of the biomarker candidates in CTCs isolated from BC patients. The analyzed cohort included 60 newly diagnosed patients with BC, of which the CTCs (53 patients) or biopsies (8 patients) were evaluated. For one patient, it was possible to collect both the CTCs and biopsy. The median age of the enrolled patients was 74 years. The demographic and major clinic-pathological features of the patients enrolled in the study are summarized in Table 1. 

As shown, the BC clinical stage at the time of diagnosis ranged from the Tx to T4 stage. However, the majority of the patients had Ta stage disease. None of the patients had a distal metastasis, nor were positive nodes found at diagnosis. Recurrence was recorded in 41.4% patients. The mean follow-up period was 17.6 months, with a median of 16.8 months. 

**Figure 3:**
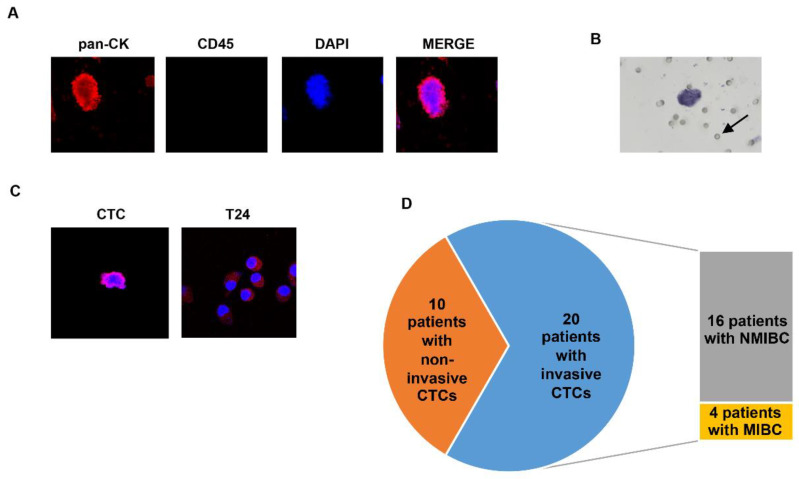
The characterization and functionality of the CTCs from BC patients. (**A**) The representative confocal microscopy image of the isolated CTCs stained with anti-human pan-CK and anti-human CD45 Abs. DAPI was used to counteract the nuclei. Magnification 60×. (**B**) The representative light microscopy image of the CTCs, stained with toluidine blue, migrated on the membrane to the bottom face. The arrow indicates the pores of the transwell. (**C**) The representative confocal image of the CTCs and T24 cells that invaded the Matrigel in the transwell invasion assay. Cells were stained with pan-CK and DAPI, Magnification 60×. (**D**) The percentage of patients who displayed CTCs with an invasive phenotype, as demonstrated by the transwell assay.

The blood samples were processed by ScreenCell devices and the CTCs were detected in all patients with the exception of three. The isolated CTCs were characterized for the expression of pan-CK and for the absence of CD45, as previously described [45] (Figure 3A). In addition, the cell invasion assay was used to investigate the viability and functionality of the isolated CTCs. For this purpose, CTCs enriched from 30 patients, of which 23 were NMIBC and seven were MIBC, were evaluated by using the in vitro invasion assay. Our results showed that the CTCs from 20 BC patients (66.7%) spread into the Matrigel, indicating an invasive and functional phenotype (Figure 3B,C). T24 BC cells, known to be high-grade and invasive, were used as the positive control [46]. Interestingly, we found that the group of invading cells was composed of CTCs derived from both the NMIBC (16 patients) and MIBC patients (four patients), suggesting that the dissemination in the blood of cells, potentially able to invade tissue, occurs very early during BC progression (Figure 3D).

**Figure 4:**
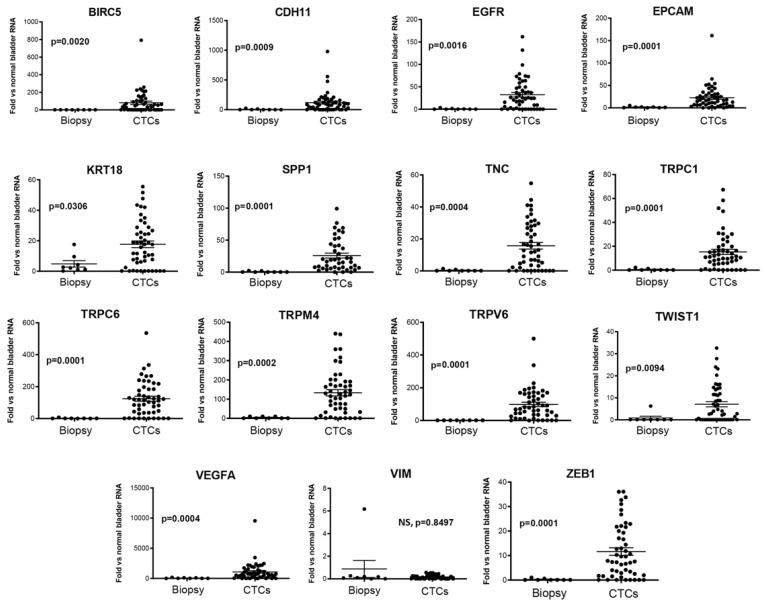
The expression of biomarker candidates in the CTCs isolated from BC patients. The expression was evaluated by ddPCR compared with the NHB and BC biopsies. Genes found to be expressed at significant higher/lower levels in CTCs with respect to the BC biopsies or NHB, are shown. The gene expression levels, evaluated by ddPCR, are expressed as fold changes with respect to NHB, used as calibrators (=1). NS = not statistically significant, *p* ≤ 0.05 was considered as statistically significant.

Then, through the ddPCR, we analyzed the expression of the selected markers in isolated CTCs. BIRC5, CDH11, EGFR, EPCAM, KRT18, SPP1, TRPA1, TRPC1, TRPC3, TRPC6, TRPV6, TRPM4, TWIST1, TNC, VEGFA, VIM, and ZEB1 genes were heterogeneously expressed. Furthermore, we compared the gene expression levels obtained in the CTCs, evaluated as fold changes with respect to the normal human bladder RNAs (NHB), with biopsies. A significantly increased expression of BIRC5, CDH11, EGFR, EPCAM, KRT18, SPP1, TNC, TRPC1, TRPC6, TRPM4, TRPV6, TWIST1, VEGFA, and ZEB1 was found in the CTCs with respect to NHB and, even more interestingly, compared with the biopsies (Figure 4 and Figure S1). 

Neither TRPA1 nor TRPC3 expression levels differed statistically from NHB or the biopsies (data not shown), so we excluded them from further analysis. No cells with CTC-like features were found in the peripheral blood of healthy donors, whose WBCs were similarly analyzed, demonstrating the negligible expression of these genes (data not shown). 

In addition, the positive detection of BIRC5, CDH11, EGFR, SPP1, TNC, TRPC6, TRPM4, TRPV6, TWIST1, VEGFA, and ZEB1 was more present in the CTC group compared with the biopsy group (Table 2). 

Overall, these data indicate that the increase in the expression levels of several genes involved in the processes of angiogenesis, epithelial–mesenchymal transition (EMT), and cation flux–regulation pathways is associated with the acquisition of the CTC phenotype.

### 3.3. Biomarkers Predictive of Recurrence

**Figure 5:**
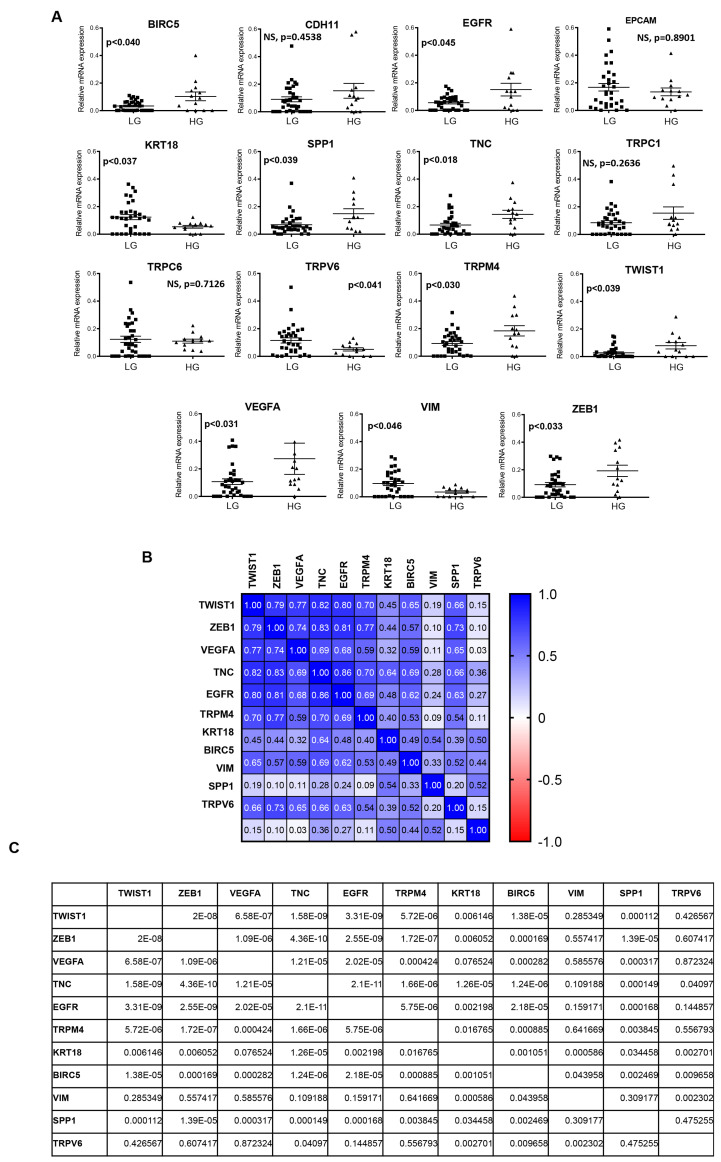
The gene expression analysis of the selected biomarkers. (**A**) The analysis was performed in the CTCs by stratifying patients according to high grade (HG) and low grade (LG). The Mann–Whitney t-test. *p* ≤ 0.05 was considered as statistically significant. (**B**,**C**) The Spearman correlation matrix of the selected biomarker candidates. NS = not statistically significant, *p* ≤ 0.05 was considered as statistically significant.

Then, to investigate the relationship between the marker gene expression levels in CTCs and tumor stage, we analyzed the mRNA expression levels of BIRC5, CDH11, EGFR, EPCAM, KRT18, SPP1, TRPC1, TRPC6, TRPV6, TRPM4, TWIST1, TNC, VEGFA, VIM, and ZEB1 in the CTCs from the NMIBC and MIBC patients (Figure S2). Our data showed no statistically significant differences by comparing the NMIBC with MIBC patients. In addition, to investigate the association between the molecular profile and histological classification, we analyzed the expression of the selected markers in the CTCs by stratifying the BC patients according to high (grade HG) and low grade (LG). Our results demonstrated that BIRC5, EGFR, SPP1, TNC, TRPM4, VEGFA, TWIST1, and ZEB1 were upregulated whereas KRT18, TRPV6, and VIM were downregulated in the HG with respect to the LG BC (Figure 5A). No differences were found in the expression levels of CDH11, EPCAM, TRPC1, and TRPC6, thus they were excluded in the following analysis. This result prompted us to evaluate the correlation index in order to strengthen the relationship between the biomarkers according to their expression levels in the CTCs. The data established the presence of two well-defined clusters of strong association: one group consisting of BIRC5, EGFR, KRT18, SPP1, TNC, TWIST1, TRPM4, VEGFA, and ZEB1 genes and the second of TRPV6 and VIM, suggesting a suitable gene model for CTCs from BC patients (Figure 5B,C and Figure S3). 

### 3.4. Identification of a Four-mRNA Based Signature

Through the univariate Cox analysis, we analyzed whether the biomarker expression levels of the two clusters were associated with the prognosis of BC patients. Our results showed that the RFS was significantly associated with the expression levels of EGFR, TRPM4, TWIST1, and ZEB1 belonging to the same cluster. These data outline a four-mRNA-based model to predict the RFS time in BC patients (Table 3).

**Figure 6:**
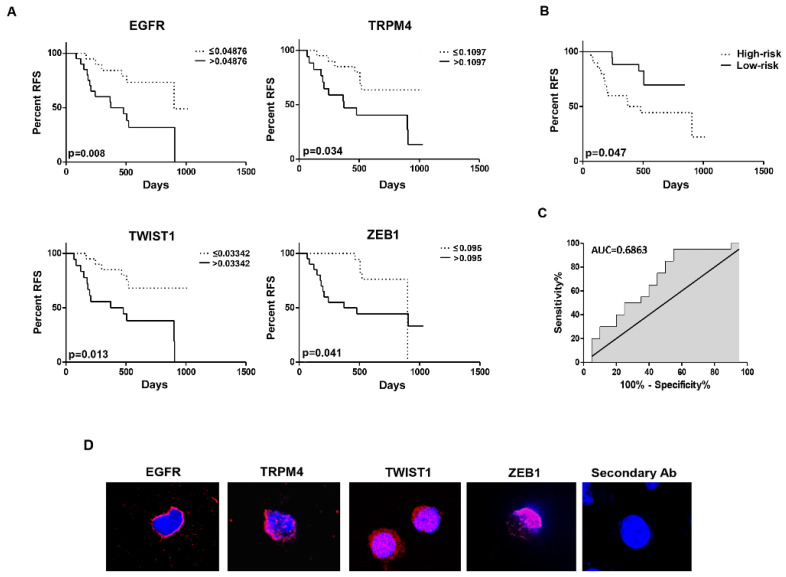
The development of the four-mRNA-based prognostic model. (**A**) The Kaplan–Meier analysis for RFS by stratifying patients into high- and low-expressing for the EGFR, TRPM4, TWIST1, and ZEB1 levels. (**B**) The Kaplan–Meier and (**C**) ROC curve analysis for RFS by stratifying patients into the high- and low-risk score according to the four-mRNA-based signature. (**D**) The representative images of CTCs stained with anti-human EGFR, TRPM4, TWIST1, and ZEB1 Abs followed by Alexa 594-conjugated secondary Ab. DAPI was used to counteract the nuclei. Magnification 60×.

Furthermore, Kaplan–Meyer analysis was used to validate the association between the expression of four gene candidates and the prognosis of patients. For this purpose, first, we stratified patients into high- and low-expressing, according to the ROC analysis, for the EGFR, TRPM4, TWIST1, and ZEB1 markers. As shown in Figure 6A, patients expressing high levels of EGFR, TWIST1, TRPM4, and ZEB1 had worse RFS compared with those that had low levels. Profiles retrieved from the GTEx showed the insignificant expression of the four selected biomarkers in whole blood with respect to the bladder, making our analysis consistent and reliable (Figure S4).

### 3.5. The Risk Score Based on Four-mRNA Signature Predicted the RFS of BC Patients: Validation of the Prognostic Model

To facilitate the application of identified RFS-related mRNAs in the clinical management of BC patients, the risk score for each patient was constructed with the regression coefficient from the univariate Cox analysis by using: Risk score = (3.002 × Expression EGFR + 4.514 × Expression TRPM4 + 5.360 × Expression TWIST1 + 3.590 × Expression ZEB1). Then, the patients were stratified into high- and low-risk groups according to the median risk score, which was used as the cutoff point. The Kaplan–Meier analysis showed a shorter RFS in the high-risk group with respect to the low-risk group (Figure 6B) as also supported by the ROC curve, reaching an AUC value of 0.6863 (Figure 6C). Overall, these results demonstrated that the risk score displayed a substantially effective performance for RFS prediction and it can be used as a prognostic indicator. Confocal microscopy was also used to assess the expression at the protein level of the four biomarkers. The data showed that the CTCs expressed at the protein levels the four markers EGFR, TRPM4, TWIST1, and ZEB1 (Figure 6D). Finally, multivariate analysis, by considering the risk score, grade, stage, diabetes type II, hypertension, and RFS, showed that the four-mRNA-based signature was an independent prognostic factor in BC patients (Table 4).

## 4. Discussion

The lack of an accurate classification system predicting the risk of recurrence or the progression of BC patients hinders the search of new biomarkers. Indeed, even though TURBT is the standard of care for the management of BC [47], its main purpose is quite different in NMIBC and MIBC, and NMIBC patients still have a heterogeneous prognosis [48]. The fact that the prognosis for BC patients has not changed for over three decades [49], and the identified molecular markers are rarely discriminatory enough for clinical use [50,51] suggests the need of a deeply understanding if this pathology.

In the last few years, most of the studies have focused their attention on liquid biopsy and the enrichment of CTCs, also in BC [18,52,53]. In particular, the number of CTCs detected in the peripheral blood of BC patients has been considered as a prognostic indicator of cancer prognosis [25,54]. The presence of CTCs predicting decreased time to first recurrence and time to progression identified a subgroup of patients with super-high-risk CTC-positive NMIBC. There was a non-statistical difference in the number of preoperative CTCs between the NMIBC and MIBC patients, suggesting that further investigation is needed [26].

The spread of CTCs in the bloodstream represents a fundamental moment for the spread of cancer, even if it is now accepted that only a small percentage of these cells can survive in the circulation and therefore give rise to metastases [55]. In line with these previous findings, we demonstrated that CTCs, obtained by size-based isolation, are present in the peripheral blood of BC patients. Interestingly, we also showed that the isolated CTCs from the NMIBC and MIBC patients displayed the same in vitro invasive ability. This indicates that their dissemination occurs very early during BC progression and that CTC features are independent from the phenotype of the primary tumor counterpart, underlying the importance to better explore CTCs as a source of potential prognostic biomarkers that are useful immediately after the diagnosis.

Thus, our aim was to identify a specific CTC gene expression profile correlated to the ability of recurrence. First, we analyzed several potential markers such as BIRC5, CDH11, EPCAM, KRT18, SPP1, TIMP2, UPK2, TRPA1, TRPC1, TRPC3, TRPC6, TRPV6, TRPM4, TWIST1, TNC, VEGFA, VIM, and ZEB1 disclosed as related to the BC primary tumor in studies published back to 2010. The expression levels of these biomarker candidates were then analyzed in the CTCs from BC patients. No differences were found in the expression by comparing the CTCs from the NMIBC with MIBC patients whereas several markers were expressed at different levels in HG with respect to the LG samples. For this reason, we investigated them in order to evaluate the relation with RFS. The univariate analysis, also supported by the Kaplan–Meyer, showed that in our study including the NMIBC and MIBC patients, four biomarkers—EGFR, TRPM4, TWIST1, and ZEB1—are strongly associated with RFS.

Our data were in line with recent findings that describe the association between high EGFR expression and shorter RFS, not only in the MIBC, but also in stage T1 NMIBC patients, suggesting early stratification is essential in order to select the most appropriate targeted treatment [56]. Encouraging results have been achieved by using small molecules targeting EGFR such as erlotinib [57]. In fact, EGFR signaling regulates numerous cellular pathways associated with tumor invasion and metastasis in preclinical models of BC. Moreover, recently, the targeted toxin EGF-PE40, obtained by combining the ligand EGF with PE40, a truncated version of Pseudomonas Exotoxin A, was generated and the expression of EGFR in BC cells was used to internalize the toxin and promote cell death [58].

The TRPM4 channel is a non-selective monovalent cationic channel expressed in several tissues including smooth muscle and urothelial cells of bladder. The TRPM4 activation leads to sodium ion influx into the cell with consequent plasma membrane depolarization and calcium signaling [59]. It has been suggested that TRPM4 has a key role in normal bladder contraction. However, the absence of information about its involvement in urothelial cancer suggests that further efforts are needed to understand its physiological and pathological functions. On the other hand, it is well-known that TRPM4 is involved in the growth and progression of several cancers. In fact, germline variants of TRPM4, affecting the intestinal mucosal integrity, have been found in colon rectal cancer cells [60]. Moreover, TRPM4 is responsible for stemness mediation in breast cancer. It is overexpressed in breast cancer tumor-spheres and its inhibition displays anti-tumor effects by directly targeting cancer stem cells [61]. Finally, it has also been classified as a cancer driver gene in androgen-independent prostate cancer where it regulates the EMT, which is fundamental for cancer cell migration and invasion. Therefore, given the numerous associations with cancer hallmark functions, TRPM4 has been suggested to be a new potential anticancer drug target [62].

TWIST1 overexpression is frequently detected in several cancers also including BC [63]. Its expression has been associated with the invasive aggressive cancer behavior, recurrence, therapy failure, and poor prognosis for the patient. In fact, it is well-accepted that it is involved in tumor initiation, intravascular migration, EMT, metastasis, and angiogenesis [64]. In addition, it has been demonstrated in BC that TWIST1, by regulating the expression of ABC transporters and the AKT signaling, induces resistance to several chemotherapeutic drugs [63]. Finally, the expression of TWIST1 was significantly enhanced in the metastatic lesions with respect to the BC primary site, consolidating its pivotal role in the metastatic process [65]. Thus, our data, obtained by using CTCs from NMIBC and MIBC, show the inclusion of TWIST1 in the four-based gene signatures, are in agreement with these previous findings, which support the potential diagnostic and prognostic role of TWIST1 in BC [45,65].

Finally, ZEB1, the fourth biomarkers identified in our study, was significantly overexpressed in BC in comparison with the healthy adjacent tissues. Moreover, it is associated with vasculogenic mimicry and with EMT promotion in BC [66]. In fact, its knockdown markedly reduced the formation of vasculogenic mimicry in the BC cell lines. Recently, it has also been demonstrated that the VIM Antisense RNA 1/miR-655/ZEB1 axis regulates metastasis in BC by modulating the EMT. In fact, VIM Antisense RNA 1, by competing with ZEB1 for miR-655 binding, abolishes the miR-655-mediated suppression of ZEB1 that is then able to finally affect EMT [67]. Our results are supported by findings described in breast cancer cells, where ZEB1 expression plays a pivotal role in the maintenance of stem-like features, immune evasion, epigenetic reprogramming, and aberrant cellular polarity [68]. Overall, these findings strongly highlight the implication of ZEB1 in the resistance and survival of disseminated cancer cells.

Identifying the features of primary tumors is insufficient to manage the disease’s progression, as single-site tumor biopsies cannot accurately portray the extremely variable cell population in the tumor mass. In this regard, CTCs are found to be genetically different and subjected to a distinct evolutionary process than the primary tumor mass. For this reason, the non-invasive liquid biopsy and the examination of CTCs could represent a good choice to replace the tissue biopsy in order to better evaluate the optimal therapy and predict the prognosis of the patients [69].

## 5. Conclusions

The results from this study demonstrated that the expression of the four-gene signature in CTCs was markedly associated with recurrence, suggesting an innovative strategy to manage disease progression without repetitive invasive procedures.

In the near future, the precise stratification of patients based on the EGFR, TRPM4, TWIST1, and ZEB1 gene signature risk score obtained in CTCs could represent, immediately after the diagnosis, a strategy to better plan the clinical evolution of the patient and therefore select the best therapeutic management. Indeed, faster follow-up, early radical surgery, or systemic therapy may be approved in the very high-risk group of patients, leading to the possibility of reducing the number of cystoscopies performed that are onerous for patients and expensive for health care providers. However, we believe that it is urgent to conduct studies with larger cohorts of patients to validate this risk score in the clinical practice.

## Figures and Tables

**Table 1:** The baseline clinico-pathologic characteristics in the prospective study of patients with BC.

Variable	Patients
All, *n* (%)	60 (100.0%)
Gender, *n* (%)	
Male	52 (86.7%)
Female	8 (13.3%)
Age, years	
Range	47–93
Median	74
Tumor grade, *n* (%)	
Low	45 (75.0%)
High	15 (25.0%)
Tumor stage, *n* (%)	
Tx	3 (5.0%)
Tis	4 (6.7%)
Ta	37 (61.75)
T1	8 (13.3%)
T2	4 (6.7%)
T3	3 (5.05)
T4	1 (1.65)
Histology	
Papillary	34 (56.6%)
No papillary	26 (43.4%)
CTC status	
Negative	3 (5.6%)
Positive	50 (94.4%)
Local Recurrence	
No	35 (58.3%)
Yes	25 (41.7%)
Infiltrated nodes	
No	60 (100%)
Yes	0 (0%)
Metastasis	
No	60 (100%)
Yes	0 (0%)
Diabetes type II	
Yes	18 (30%)
No	42 (70%)
Hypertension	
Yes	29 (48.3%)
No	31 (51.7%)

**Table 2:** The analysis by the Fisher’s exact test of the marker expression in the CTCs with respect to the biopsies.

Markers	Biopsy *n* (%)	CTC *n* (%)	*p*-Value
BIRC5	0 (0.0)	32 (64.0)	* *p* = 0.0008
CDH11	2 (25.0)	38 (76.0)	* *p* = 0.0082
EGFR	3 (37.5)	38 (76.0)	* *p* = 0.0082
EPCAM	8 (100.0)	46 (92.0)	NS, *p* > 0.9999
KRT18	7 (87.5)	40 (80.0)	NS, *p* > 0.9999
SPP1	2(25.0)	40 (80.0)	* *p* = 0.0001
TNC	2 (25.0)	39 (78.0)	* *p* = 0.0057
TRPC1	4 (50.0)	41 (82.0)	NS, *p* = 0.0660
TRPC6	4 (50.0)	42 (84.0)	* *p* = 0.0489
TRPV6	0 (0.0)	45 (90.0)	* *p* < 0.0001
TRPM4	3 (37.5)	42 (84.0)	* *p* = 0.0105
TWIST1	1 (12.5)	32 (64.0)	* *p* = 0.0161
VEGFA	4 (50.0)	42 (84.0)	*p = 0.0489
VIM	7 (87.5)	36 (72.0)	NS, *p* = 0.6660
ZEB1	2 (25.0)	42 (84.0)	* *p* = 0.0016

Abbreviations: BIRC5—baculoviral IAP repeat containing 5; CDH11—cadherin 11; CTC—circulating tumor cell; EGFR—epidermal growth factor receptor; EPCAM—epithelial cell adhesion molecule; KRT18—keratin 18; SPP1—secreted phosphoprotein 1; TNC—tenascin; TRPC—transient receptor potential canonical; TRPM—transient receptor potential melastatin; TRPV—transient receptor potential vanilloid; TWIST1—Twist family BHLH transcription factor 1; VEGFA—vascular endothelial growth factor A; VIM—vimentin; ZEB1—zinc finger E-box binding homeobox 1. NS = not statistically significant, * *p* ≤ 0.05 was considered as statistically significant.

**Table 3:** The univariate Cox regression analysis of the selected biomarkers and RFS.

Markers	HR (95% CI)	*p*-Value
BIRC5	2.60 (3.36 × 10^−3^–2010)	NS, 0.778
EGFR	20.120 (1.01–419.4)	0.050 *
KRT18	0.049 (5.28 × 10^−04^–4.58)	NS, 0.193
SPP1	8.46 (0.60–118.2)	NS, 0.113
TNC	24.49 (0.13–4681)	NS, 0.233
TRPM4	91.33 (1.02–8944)	0.050 *
TRPV6	0.28 (7.35 × 10^−04^–105.3)	NS, 0.673
TWIST1	212.89 (5.43–8348)	0.005 *
VEGFA	17.83 (0.78–406.4)	NS, 0.071
VIM	5.17 × 10^−3^ (1.42 × 10^−05^–1.88)	NS, 0.080
ZEB1	33.42 (1.25–891.3)	0.036 *

Abbreviations: BIRC5—baculoviral IAP repeat containing 5; CI—confidence interval; EGFR—epidermal growth factor receptor; KRT18—keratin 18; HR—hazard ratio; SPP1—secreted phosphoprotein 1; TNC—tenascin; TRPM—transient receptor potential melastatin; TRPV—transient receptor potential vanilloid; TWIST1—Twist family BHLH transcription factor 1; VEGFA—vascular endothelial growth factor A; VIM—vimentin; ZEB1—zinc finger E-box binding homeobox 1. NS = not statistically significant, * *p* ≤ 0.05 was considered as statistically significant.

**Table 4:** The univariate and multivariate analysis of the risk score, grade, stage, diabetes type II, hypertension, and RFS.

Variables		Univariate Analysis		Multivariate Analysis	
		HR (95% CI)	*p*-Value	HR (95% CI)	*p*-Value
Grade	HG/LG	1.46 (0.40–5.32)	0.566	2.03 (0.42–9.77)	0.375
Stage	Ta-Tis-T1/T2–4	2.56 (0.33–20.1)	0.371	2.08 (0.16–27.64)	0.579
Diabetes type II	Yes/No	1.29 (0.45–3.74)	0.824	0.75 (0.23–2.42)	0.630
Hypertension	Yes/No	1.97 (0.54–7.18)	0.301	3.13 (0.72–13.53)	0.127
Risk score	High/Low	1.35 (1.03–1.76)	0.027 *	1.35 (1.03–1.77)	0.030 *

Abbreviations: CI—confidence interval; HR—hazard ratio. NS = not statistically significant, * *p* ≤ 0.05 was considered as statistically significant.

## Data Availability

The data that support the findings of this study are available from the corresponding authors upon request.

## Supplementary Materials

The following supporting information can be downloaded at: https://www.mdpi.com/article/10.3390/cancers14133118/s1. Figure S1: The gene expression of selected biomarker candidates in biopsies with respect to the NHB. Figure S2: The gene expression analysis of selected biomarkers; Figure S3: The correlation matrix analysis; Figure S4: The profiles retrieved from the GTEx to support and validate our selection.
