# Supplementary material for: The Prognostic Value of the Circulating Tumor Cell-Based Four mRNA Scoring System: A New Non-Invasive Setting for the Management of Bladder Cancer
Source: Cancers (Basel). 2022 Jun 25;14(13):3118. doi: 10.3390/cancers14133118 (PMC9264990; doi:10.3390/cancers14133118)
Supplement: Supplementary file 1 [file cancers-14-03118-s001.zip › cancers-1756305-supplementary.pdf]

Supplementary Materials

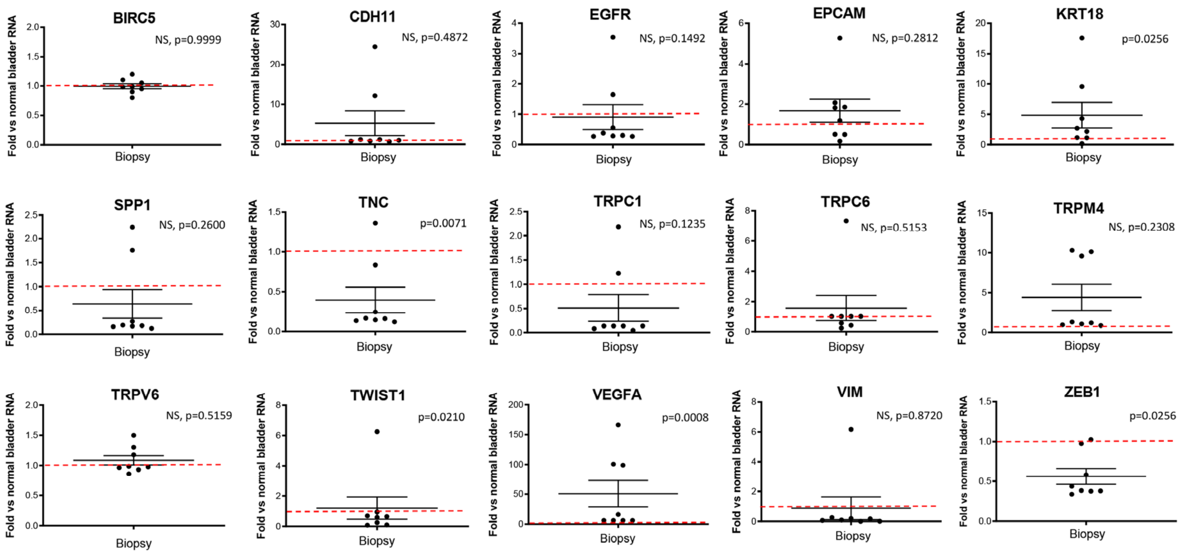

Figure S1. Gene expression of selected biomarker candidates in biopsies respect to NHB**.** Gene expression levels, evaluated by ddPCR, are expressed as fold changes with respect to NHB, used as calibrators=1. NS= not statistically significant, * $p \leq 0.05$ was considered as statistically significant. Red dotted line represents NHB used as calibrator= 1.

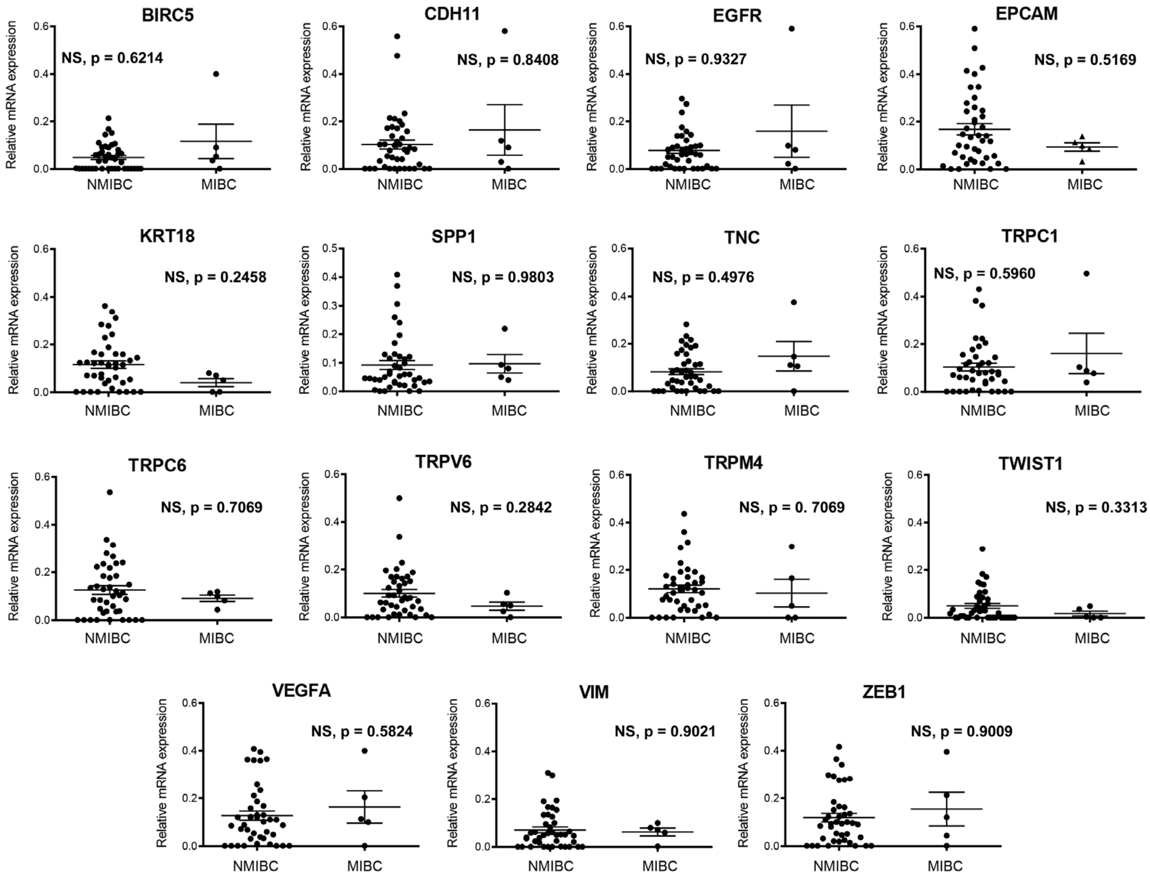

Figure S2. Gene expression analysis of selected biomarkers. The analysis was performed in CTCs by stratifying patients according to clinico-pathological subgroups in NMIBC and MIBC. Unpaired, non-parametric, Mann-Whitney test was used to analyze the significant level of the selected biomarkers between NMIBC and MIBC.

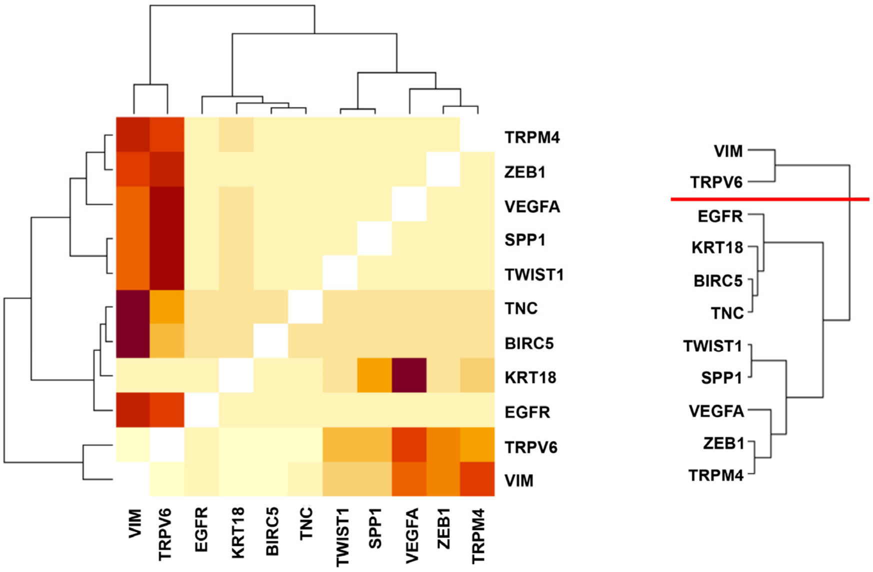

Figure S3. Correlation matrix analysis. Heat map and hierarchical tree of selected biomarker candidates based on correlation matrix values.

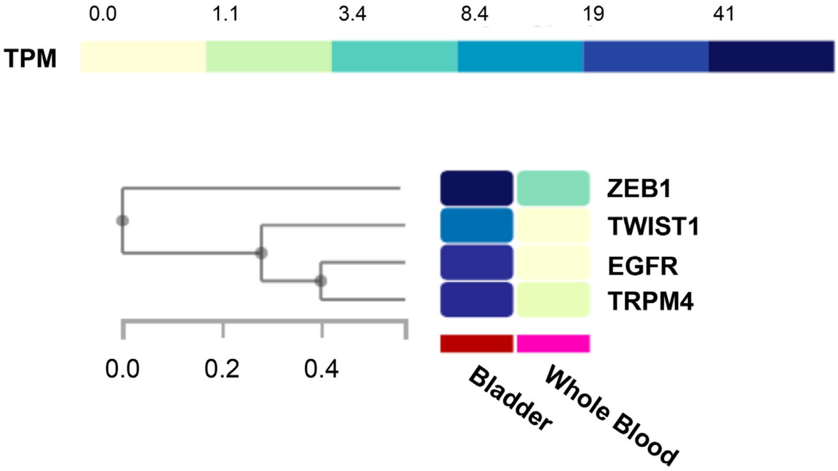

Figure S4. Profiles retrieved from the GTEx to support and validate our selection. Heatmap showing the expression level of the selected markers in bladder and whole blood.
